# Eggmanone Effectively Overcomes Prostate Cancer Cell Chemoresistance

**DOI:** 10.3390/biomedicines9050538

**Published:** 2021-05-12

**Authors:** Chen Xie, Pen-Jen Lin, Jijun Hao

**Affiliations:** 1 College of Veterinary Medicine, Western University of Health Sciences, Pomona, CA 91766, USA; cxie@westernu.edu; 2 Graduate College of Biomedical Sciences, Western University of Health Sciences, Pomona, CA 91766, USA; plin@westernu.edu

**Keywords:** chemoresistance, prostate cancer, Phosphodiesterase-4, cancer stem cells, Eggmanone

## Abstract

Prostate cancer chemoresistance is a major therapeutic problem, and the underlying mechanism is not well understood and effective therapies to overcome this problem are not available. Phosphodiesterase-4 (PDE4), a main intracellular enzyme for cAMP hydrolysis, has been previously shown to involve in the early chemo-sensitive prostate cancer cell proliferation and progression, but its role in the more-advanced chemo-resistant prostate cancer is completely unknown. Here we found that the expression of PDE4 subtype, PDE4D, is highly elevated in the chemo-resistant prostate cancer cells (DU145-TxR and PC3-TxR) in comparison to the chemo-sensitive prostate cancer cells (DU145 and PC3). Inhibition of PDE4D with a potent and selective PDED4 inhibitor, Eggmanone, effectively decreases the invasion and proliferation as well as induces cell death of the chemo-resistant prostate cancer cells (DU145-TxR and PC3-TxR). These results were confirmed by siRNA knockdown of PDE4D. We and colleagues previously reported that Eggmanone can effectively blocked sonic Hedgehog signaling via PDE4D inhibition, and here our study suggests that that Eggmanone downregulated proliferation of the chemo-resistant prostate cancer cells via sonic Hedgehog signaling. In addition, Eggmanone treatment dose-dependently increases docetaxel cytotoxicity to DU145-TxR and PC3-TxR. As cancer stem cells (CSCs) are known to be implicated in cancer chemoresistance, we further examined Eggmanone impacts on CSC-like properties in the chemo-resistant prostate cancer cells. Our study shows that Eggmanone effectively down-regulates the expression of CSCs’ marker genes Nanog and ABC sub-family G member 2 (ABCG2) and attenuates sphere formation in DU145-TxR and PC3-TxR cells. In summary, our work shows that Eggmanone effectively overcomes the chemoresistance of prostate cancer cells presumably through sonic Hedgehog signaling and targeting CSCs, suggesting that Eggmanone may serve as a novel agent for chemo-resistant prostate cancer.

## 1. Introduction

Chemoresistance is a major therapeutic problem for patients with prostate cancer, the second most frequent malignancy in men worldwide [1]. The early-stage prostate cancer is androgen-sensitive and androgen-deprivation therapy (ADT) is often effective initially [2,3,4]. However, the disease eventually progresses to androgen-independent prostate cancer, also known as castration-resistant Prostate cancer (CRPC). The drug docetaxel (Taxotere) is the mainstay chemotherapy for patients with metastatic CRPC. However, almost inevitably, patients treated with docetaxel will develop chemoresistance [5]. More recently, the combination of docetaxel with ADT therapy to treat the early-stage androgen-sensitive prostate cancer has shown overall improved outcome [6,7]. Unfortunately, this combination therapy does not stop the prostate cancer progression to the chemoresistance, and early administration of docetaxel at the androgen-sensitive prostate cancer stage may antedate the onset of the chemoresistance [8,9]. Currently, the molecular mechanism for the prostate cancer chemoresistance is poorly understood, and effective therapies to overcome this problem are not available.

Phosphodiesterase-4 (PDE4) is a main intracellular enzyme for cAMP hydrolysis and plays a major role in cellular signaling. PDE4 consists of four subtypes, PDE4A, PDE4B, PDE4C and PDE4D, and is involved in several human malignancies including prostate cancer, leukemia, colon cancer and glioma [10,11,12,13,14]. For instance, PDE4A expression is elevated in central nervous system tumor cells [12,15], and PDE4B expression in increased in hematologic malignancies [16,17], whereas hypermethylation of PDE4C promoter sites was reported in high-grade glioma samples [18]. In addition, overexpression of PDE4D was reported in human CRPC prostate adenocarcinoma samples in comparation with benign prostatic hyperplasia samples [11], and knockdown or inhibition of PDE4D reduces the growth of both CRPC prostate cancer cells in vitro and in vivo [11,19]. However, to date, no studies of PDE4 in the more-advanced chemo-resistant prostate cancer have been reported.

We and colleagues have previously reported that Eggmanone, a selective allosteric PDE4D inhibitor, specifically targets the upstream conserved region 2 (UCR2) of PDE4D, instead of its catalytic domain [20], which is believed to overcome severe emesis and other side effects associated with current PDE4 competitive inhibitors [21,22,23]. Moreover, Eggmanone was picked up in an in vivo zebrafish embryo-based screening [20], suggesting it potentially has desired drug-like properties. Furthermore, our recent study indicated that Eggmanone displays favorable drug pharmacokinetics in rats [24].

In this study, we examined PDE4 expression in chemo-sensitive and chemo-resistant prostate cancer cells and identified PDE4D is only subtype of PDE4 that shows enhanced expression in chemo-resistant prostate cancer cells in comparison to chemo-sensitive prostate cancer cells. We further demonstrated that PDED4 inhibitor, Eggmanone, effectively overcomes the chemo-resistance of prostate cancer cells.

## 2. Materials and Methods

### 2.1. Cell Culture and Reagents

The human androgen-independent prostate cancer cell lines DU145 and PC-3 were obtained from American Type Culture Collection (ATCC, Manassas, VA, USA). They were cultured in RPMI 1640 supplemented with 10% FBS (Gibco) and 1% penicillin/streptomycin (GenClone^®^) in an atmosphere of 5% CO_2_ at 37 °C. Docetaxel and Eggmanone were purchased from Sigma-Aldrich Co. (St Louis, MO, USA). The docetaxel resistant cell lines (PC3-TxR and DU145-TxR) which were established using a stepwise increase in exposure to docetaxel were kindly provided by Moses Chow, Western University of Health Sciences, Pomona, CA, USA.

### 2.2. Cell Scratch-Wound Assay

PC3, DU145, PC3-TxR and DU145-TxR cells were seeded in 35 mm dishes to create a confluent monolayer. The dishes were allowed to incubate overnight in order to allow the cells to attach to the bottom of the dish. On the following day, wounds were created by a straight scratch from a pipette tip in the center of the culture. The cells were then treated with DMSO, docetaxel (1 nM), Eggmanone (3 µM) or the combination of docetaxel (1 nM) and Eggmanone (3 µM), respectively. Photographs were taken when wounds were created and after 22 h’s incubation using phase-contrast microscopy, and gap distances were quantitatively evaluated using software ImageJ. The gap distances after 22 h’s incubation were normalized with the gap distance at 0 h as the migration rates.

### 2.3. Cell Proliferation Assay

The cell proliferation was evaluated by a Colorimetric Method (MTS Assay) using CellTiter 96^®^ AQueous One Solution Cell Proliferation Assay (Promega, Madison, WI, USA). Briefly, cells were plated in 96-well plate at a concentration of 8000 cells per well. The cells were incubated at 37 °C in a 5% CO_2_ incubator overnight followed by different treatments for 72 h. Then 20 μL of CellTiter 96^®^ AQueous One Solution Reagent was added into each well of the 96-well assay plate containing the samples in 100μL of culture medium for 1 h. The absorbance was read at 490 nm on a POLARstar spectrophotometer (BMG Labtech, Cary, NC, USA). The results were expressed as percentage of treated cells compared to untreated control using the equation: Viable% = Absorbance(test)/Absorbance(control) × 100). All the readings were normalized to the control and the control was considered 100%. The IC50 was calculated using an Emax sigmoid model with the aid of GraphPad Prism software (GraphPad Prism 8.3.1, San Diego, CA, USA). The experiments were performed in triplicate for each treatment group.

### 2.4. Transfection

For the knockdown experiments, siRNA control (cat#: AM4611, Life Technologies Corporation) and siRNA PDE4D (cat#: AM16708, Life Technologies Corporation, Carlsbad, CA, USA) were transfected into the DU145-TxR and PC3-TxR cells, respectively, with Fugene HD transfection reagent (Promega, Madison, WI, USA) according to the manufacturer’s instructions.

### 2.5. Cell Apoptosis Assay

CellEvent™ caspase-3/7 green detection reagent (Themo Fisher, Waltham, MA, USA) was used for cell apoptosis assay. In brief, after 24-h treatment, the cell culturing media were replaced with 5% FBS in PBS buffer supplemented with a final concentration of 3 μM caspase-3/7 green detection reagent. After 1-h incubation, the cells were then fixed with 4% formaldehyde for 15 min at room temperature, followed by another 10 minutes of incubation with 0.2% Triton X-100 in PBS buffer at 4 °C. Then 1 μg/mL DAPI was added for counterstaining. The images were taken by using a fluorescence microscopy with absorption/emission maxima of ~502/530 nm (EVOS FL, Thermo Fisher Scientific, Waltham, MA, USA).

### 2.6. Modified Boyden Chamber Invasion Assay

Cell invasion was measured using a 24-Multiwell Insert System (8 µm membrane, GenClone^®^) according to the manufacture instruction. The cell culture inserts were coated with Matrigel (BD Biosciences, San Jose, CA, USA). The cells were seeded at a concentration of 1 × 10^5^ cells/chamber. After 72-h incubation with or without different treatments, cells that had not moved to the lower wells were removed from the upper face of the filters using cotton swabs. The transwell inserts were fixed in 4% PFA for 10 min followed by staining in 0.2% crystal violet for 5 min. Then the cells that invaded through the Matrigel-coated-inserts were counted. Mean values for three randomly selected fields were obtained for each well. Experiments were performed in duplicate. Mean values for three random fields were obtained for each well.

### 2.7. Sphere Formation Assay

Cells were plated at 1000 cells/mL on low-attached 6-well-plate for suspension culture. Cells were grown in serum free Prostate Epithelial Cell Growth Basal Medium (Lonza Walkersville, MD, USA) supplemented with 4 µg/mL insulin (Sigma-Aldrich, St Louis, MO, USA), B27 (Thermo Fisher Scientific, Waltham, MA, USA), 20 ng/mL basic fibroblast growth factor (bFGF; Sigma-Aldrich), 20 ng/mL epidermal growth factor (EGF; Sigma-Aldrich, St Louis, MO, USA) for 14 days. The sphere-forming capacity was assessed by the number of colonies and the sphere size larger than 50 µm diameter was calculated. The Images were taken using an EVOS FL microscope (Thermo Fisher Scientific, Waltham, MA, USA).

### 2.8. Real-Time PCR

RNA was extracted by resuspending the cells in lysis buffer and purified by filtration following the manufacturer’s protocol (RNeasy mini Kit, Qiagen, Hilden, Germany). The first-strand cDNAs were synthesized using the High-capacity cDNA Reverse Transcription kit (Applied Biosystems) according to the manufacturer’s instructions. Using cDNA as template, real-time (RT)-PCR reactions were carried out using Fast Syber Green (2×) Master Mix Applied Biosystems). The reactions were performed in triplicate on Bio-Rad CFX connected Real-Time PCR system. Human glyceraldehyde-3-phosphate dehydrogenase (GAPDH) gene was used as an internal control. The primer sets used in this study are shown in Supplementary Table S1.

### 2.9. Western Blotting

Cells were lysed with RIPA buffer (Sigma-Aldrich, St Louis, MO, USA) containing protein inhibitors (complete ULTRA Tablets, Roche) and phosphatase inhibitors (PhosSTOP, Sigma-Aldrich, St Louis, MO, USA). Samples were denatured by incubating at 95 °C for 5 min in sample buffer and separated by using SDS-PAGE gels). Then the samples were transferred to a PDVF membrane (Millipore, Burlington, MA, USA). The membrane was blocked with Odyssey Blocking solution (Li-Cor Biosciences, Lincoln, NE, USA) for 1 h at room temperature, followed by primary antibody incubation at 4 °C overnight. The primary antibodies used in the present study included rabbit anti-PDE4D (Cell Signaling Tech, Danvers, MA, USA), mouse anti-beta actin (Cell Signaling Tech, Danvers, MA, USA). The proteins were detected by Odyssey system (Li-Cor bioscience, Lincoln, NE, USA) followed by the secondary antibodies including IRDye 680-conjugated goat anti-rabbit IgG (Li-Cor Bioscience) and IRDye 800CWconjugated goat anti-mouse IgG (Li-Cor Bioscience, Lincoln, NE, USA).

### 2.10. Statistical Analysis

All values are expressed as means ± SEM. Comparison of means was conducted using Student’s t test or Graphpad Prism 8.3.1 (San Diego, CA, USA), and results were considered statistically significant if the *p*-value was <0.05.

## 3. Results

### 3.1. PDE4D Expression Is Highly Upregulated in Chemo-Resistant Prostate Cancer Cells

**Figure 1:**
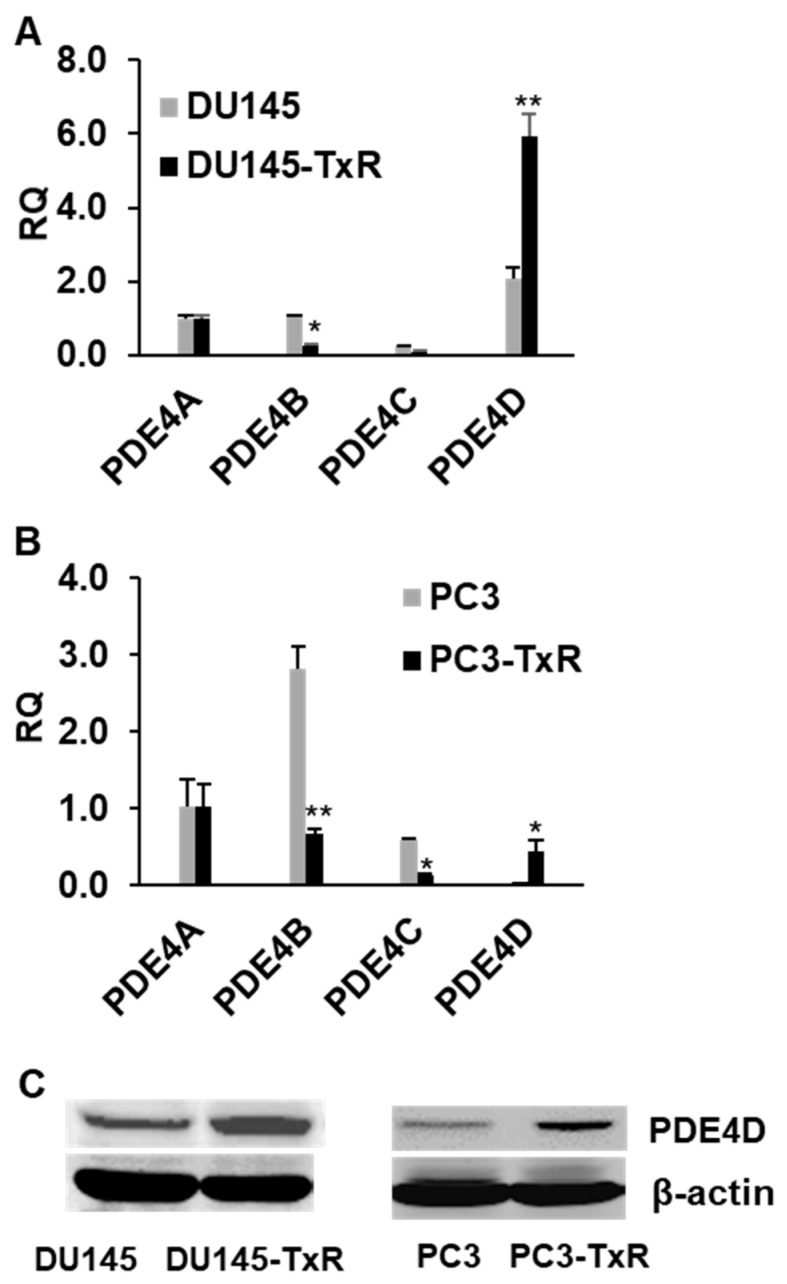
PDED4 expression is highly upregulated in chemo-resistant prostate cancer cells. (**A**) and (**B**) mRNA expression of all four PDE4 subtypes (PDE4A, PDE4B, PDE4C and PDE4D) was examined by RT-PCT in the chemo-sensitive prostate cancer DU145 and PC3 cells and the chemo-resistant prostate cancer DU145-TxR and PC3-TxR cells. The result showed that only PDE4D expression was highly upregulated in the chemo-resistant prostate cancer cell lines. RT-PC result was represented as mean relative quantity (RQ) ± SEM (*n* = 3, * *p* < 0.05, ** *p* < 0.01). (**C**) Western blotting result confirmed that PDE4D expression was elevated in the chemo-resistant prostate cancer DU145-TxR and PC3-TxR cells in comparison to the chemo-sensitive prostate cancer DU145 and PC3 cells.

PDE4 consists of four subtypes including PDE4A, PDE4B, PDE4C and PDE4D. We first examined the mRNA expression of all four PDE4 subtypes in the chemo-resistant cell lines (DU145-TxR and PC3-TxR cells) and chemo-sensitive parental cell lines (DU145 and PC3). Our RT-PCR result demonstrated that mRNA expression of only PDE4D subtype was statistically significantly enhanced in both chemo-resistant cell lines (DU145-TxR and PC3-TxR) in comparison to their chemo-sensitive parental cell lines (DU145 and PC3) (Figure 1A,B). In addition, Western blotting study further confirmed that PDE4D protein expression was dramatically elevated in chemo-resistant DU145-TxR and PC3-TxR cells in contrast to chemo-sensitive DU145 and PC3 cells (Figure 1C), implying that PDE4D expression may be associated with the prostate cancer chemo-resistance.

### 3.2. Eggmanone Decreases the Invasion of Chemo-Resistant Prostate Cancer Cells

**Figure 2:**
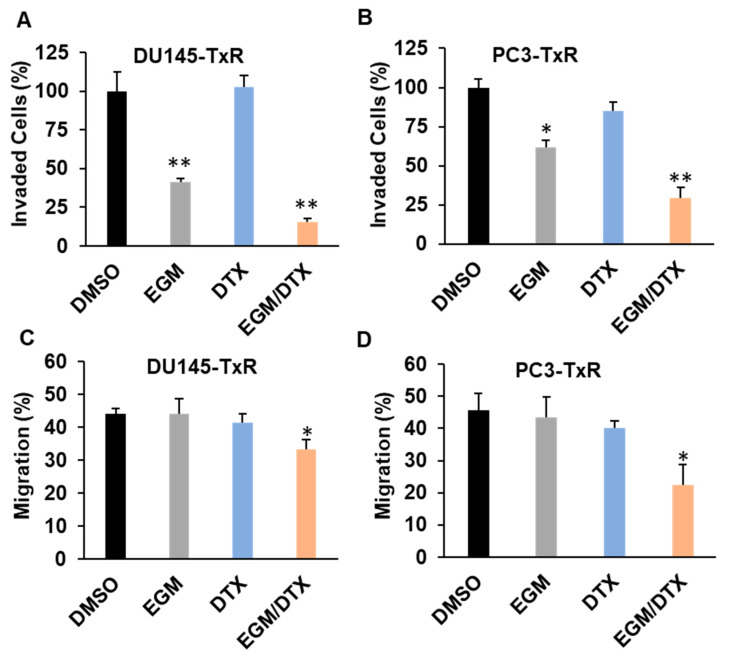
Eggmanone decreases invasion but not migration of chemo-resistant prostate cancer cells. (**A**,**B**) Effects of Eggmanone (EGM, 3 µM), Docetaxel (DTX, 1 nM) and their combination (3 µM EGM/1 nM DTX) on invasion of the chemo-resistant prostate cancer DU145-TxR and PC3-TxR cells were determined using the modified Boyden chamber assay in a 24-Multiwell Insert System (8 µM membrane, BD Biosciences) coated with Matrigel. The cells were treated for 72 h, and the invading cell percentages were normalized to the DMSO vehicle treated controls. (**C**,**D**) Effects of Eggmanone (EGM, 3 µM), Docetaxel (DTX, 1 nM) and their combination (3 µM EGM/1 nM DTX) on migration of the chemo-resistant prostate cancer DU145-TxR and PC3-TxR cells were determined using the scratch-wound assay. Cell migrations were quantified by the gap distances after 22-hr treatment normalized with the initial gap distances (*n* = 3, * *p* < 0.05, ** *p* < 0.01).

As cell invasion and migration play important roles in the progression of cancer metastasis, we examined impacts of PDE4D inhibition with Eggmanone on invasion and migration of the chemo-resistant prostate cancer cell in vitro. The cell invasion was measured by using modified Boyden chamber assay. Chemo-resistant DU-145-TxR and PC3-TxR cells were seeded on Matrigel-coated chambers, respectively, followed by 72 h incubation with DMSO, 3 µM Eggmanone, 1 nM docetaxel and the combination of 3 µM Eggmanone with 1 nM docetaxel, respectively. Eggmanone treatment dramatically reduced the cell invasion of chemo-resistant DU-145-TxR and PC3-TxR through Matrigel-coated membranes by approximately 59% and 38%, respectively, in comparison with the vehicle controls (Figure 2A,B). In addition, treatment with the combination of Eggmanone and docetaxel further decreased the cell invasion of chemo-resistant DU-145-TxR and PC3-TxR cells (Figure 2A,B). Similar result was obtained in the PDE4D knockdown with siRNA in DU145-TxR and PC3-TxR cells (Supplementary Figure S1). Effects of Eggmanone on the invasion of the chemo-sensitive prostate cancer DU145 and PC3 cells were assessed as well, and the result showed that 3 µM Eggmanone was able to reduce cell invasion by approximately 34% for DU145 cells and 27% for PC3 cells after 72 h incubation (Supplementary Figure S2A).

Next, we performed the scratch-wound assay to determine the effect of Eggmanone on the cell migration of chemo-resistant prostate cancer [25]. Chemo-resistant DU-145-TxR and PC3-TxR cells were treated with DMSO, 3 µM Eggmanone, 1 nM docetaxel and the combination of 3 µM Eggmanone with 1 nM docetaxel for 22 h, respectively, and the gap distances were then normalized with each own initial distance. The result shows that Eggmanone treatment did not slow down the migration of chemo-resistant DU145-TxR and PC3-TxR cells, but the combination of Eggmanone and docetaxel reduced their migration statistically significantly (Figure 2C,D). Eggmanone on the migration of the chemo-sensitive prostate cancer DU145 and PC3 cells were assessed as well, and the result showed that 3 µM Eggmanone did not statistically significantly reduce cell migration of DU145 and PC3 cells (Supplementary Figure S2B).

### 3.3. Eggmanone Reduces Proliferation and Induces Cell Death of Chemo-Resistant Prostate Cancer Cells

**Figure 3:**
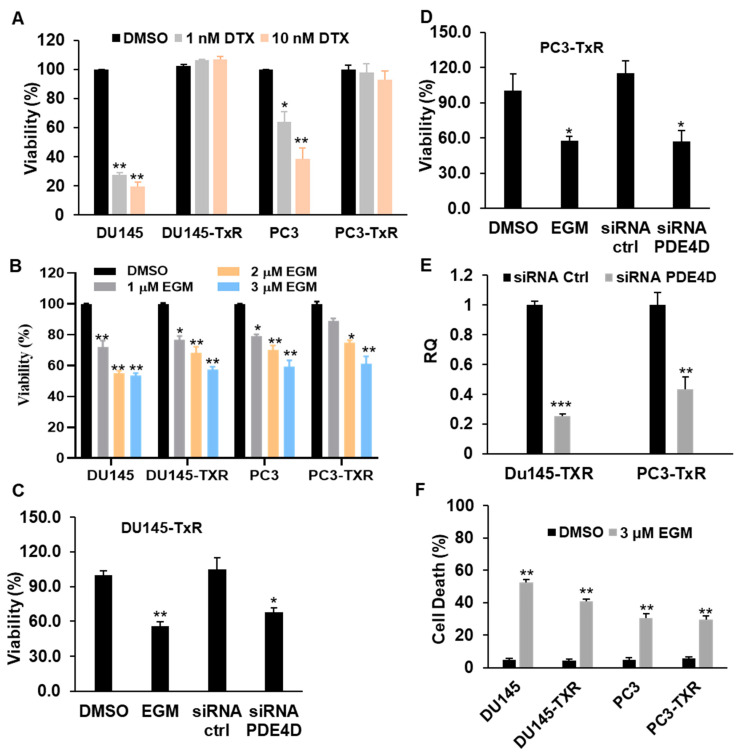
Eggmanone reduces proliferation and induces death of chemo-resistant prostate cancer cells. (**A**) The chemo-sensitive prostate cancer DU145 and PC3 cells and the chemo-resistant prostate cancer DU145-TxR and PC3-TxR cells were treated with DMSO vehicle, docetaxel at 1 nM and 10 nM concentrations for 72 h, respectively. The cell viability (%) was normalized with DMSO vehicle treated cells, and the result indicated that docetaxel treatment dramatically down-regulated cell viabilities of the chemo-sensitive DU145 and PC3 but not chemo-resistant DU145-TxR and PC3-TxR cells (up to 10 nM). (**B**) The chemo-sensitive prostate cancer DU145 and PC3 cells and the chemo-resistant prostate cancer DU145-TxR and PC3-TxR cells were treated with DMSO vehicle, Eggmanone at 1 µM, 2 µM and 3 µM concentrations for 72 h. The cell viability (%) was normalized with DMSO vehicle treated cells, and the result indicated that Eggmanone treatment dramatically down-regulated cell viabilities of both chemo-sensitive and chemo-resistant prostate cancer cells (**C**) DU145-TxR and (**D**) PC3-TxR cells were treated with DMSO vehicle, 3 µM Eggmanone, siRNA control and siRNA PDE4D, and the cell viability was assayed after 72 h. (**E**) RT-PCR result indicates that PDE4D was effectively knock downed (approximately 75% gene knockdown for DU-145-TxR and 58% for PC3-TxR). (**F**) The chemo-sensitive prostate cancer DU145 and PC3 cells and the chemo-sensitive prostate cancer DU145 and PC3 cells and the chemo-resistant prostate cancer DU145-TxR and PC3-TxR cells were treated with DMSO vehicle, Eggmanone at 3µM concentrations for 72 h, the cells were harvested for cell death assay using Trypan Blue staining. The result showed that 3 µM Eggmanone significantly reduced cell deaths in both chemo-sensitive and chemo-resistant prostate cancer cells (*n* = 3, ** *p* < 0.01). For each experiment (*n* = 3, * *p* < 0.05, ** *p* < 0.01, *** *p* < 0.001).

We examined the effect of Eggmanone on the proliferation and survival of chemo-resistant prostate cancer cells. Firstly, we performed a control experiment by treating both the chemo-sensitive cell lines (DU145 and PC3) and the chemo-resistant cell lines (DU145-TxR and PC3-TxR cells) with docetaxel for 72 h at concentrations of 1 nM and 10 nM, respectively. Then cell proliferation was determined by the MTS assay. As expected, the result showed that docetaxel dramatically reduced proliferation of the chemo-sensitive DU145 (by approximately 80% at 10 nM docetaxel) and PC3 cells (by approximately 60% at 10 nM docetaxel) (Figure 3A). In contrast, docetaxel treatment didn’t show any statistically significant effects on the proliferation of the chemo-resistant DU145-TxR and PC3-TxR cells resistant cells. All the above result demonstrated that DU145-TxR and PC3-TxR as well as their parental cell lines (DU145 and PC3) display different proliferation sensitivities to docetaxel as expected. Next, we treated these cell lines with Eggmanone for 72 h at concentrations of 1 µM, 2 µM and 3 µM, respectively. The result indicated that Eggmanone significantly suppressed the proliferation of both chemo-sensitive cells and the chemo-resistant cells in dose dependent manners (Figure 3B). For instance, 3 µM Eggmanone treatment reduced the proliferation of DU145-TxR and PC3-TxR cells by 43% and 39%, respectively, as well as suppress the proliferation of DU145 and PC3 by 46% and 42%, respectively. Next, we knock downed PDE4D with siRNA in the chemo-resistant Du145-TxR and PC3-TxR cells, and performed MTS assay after 72 h. The results showed that PDE4D knockdown, like Eggmanone treatment, statistically significantly attenuated the proliferation of the chemo-resistant Du145-TxR and PC3-TXR cells (Figure 3C,D). The RT-PCR confirmed that the PDE4D gene was effectively knocked down by siRNA in the chemo-resistant cells (Figure 3E). In summary, inhibition of PDE4D by Eggmanone or siRNA knockdown rapidly reduces the proliferation of chemo-resistant prostate cancer cells.

In addition, we examined the effect of Eggmanone on prostate cancer cell death as well. Both the chemo-sensitive cell lines (DU145 and PC3) and the chemo-resistant cell lines (DU145-TxR and PC3-TxR cells) were treated with 3 µM Eggmanone for 72 h. The floating and adherent cells were harvested followed by Trypan Blue staining to determine the number of dead and dying cells. In contrast to DMSO treatment which induced cell death in chemo-sensitive DU145 and PC3 cells by approximately 4.6% and 4.9%, respectively, Eggmanone treatment significantly increased the percentage of dead cells in DU145 and PC3 cells by 52.2% and 30.4% (Figure 3F). Similarly, in the chemo-resistant DU145-TxR and PC3-TxR cells, DMSO treatment induced cell death by approximately 4.2% and 5.7% whereas Eggmanone treatment significantly increased the percentage of dead cells by 40.8% and 29.5%, respectively, (Figure 3F). Next, we knocked down PDE4D gene in DU145-TxR and PC3-TxR cells, and then examined cell death after 72 h by Trypan Blue staining. Our result confirmed that PDE4D knock down dramatically increased cell death in DU145-TxR and PC3-TxR cells (Supplementary Figure S3). To confirm the Eggmanone-induced cell death, we performed the CellEvent™ caspase-3/7 green detection reagent (ThemoFisher) for an apoptosis assay [26]. Both DU145-TxR and PC3-TxR cells were cultured and treated with 3 µM Eggmanone or DMSO vehicle for 24 h. The culturing medium which may contain dead cells was washed away and the adherent cells were used for apoptosis assay by the CellEvent™ caspase-3/7 green detection. In consistence, the result showed that Eggmanone induced apoptosis in both DU145-TxR and PC3-TxR cells (Supplementary Figure S4). These results demonstrate that Eggmanone may be a potential agent for prostate cancer cell chemo-resistance.

### 3.4. Eggmanone Overcomes Prostate Cancer Cell Chemo-Resistance via Sonic Hedgehog Signaling

**Figure 4:**
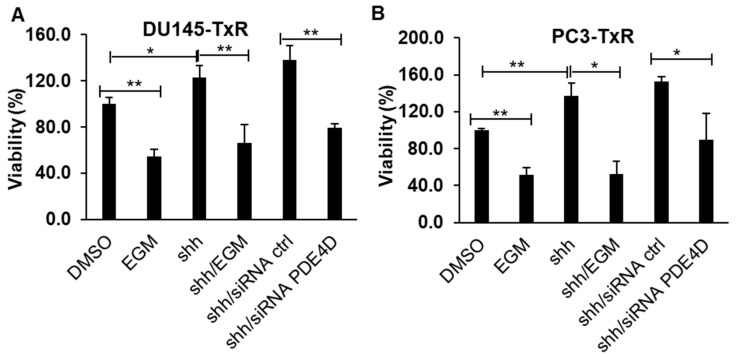
Eggmanone reduces the proliferation of chemo-resistant prostate cancer cells through sonic Hedgehog pathway. (**A**) The chemo-resistant prostate cancer DU145-TxR and (**B**) PC3-TxR cells were treated with DMSO vehicle, 3 µM Eggmanone, 200 ng/mL sonic Hedgehog (shh) ligand, 200 ng/mL shh with 3 µM Eggmanone (EGM), 200 ng/mL shh with siRNA control and 200 ng/mL shh with PDE4D knockdown with siRNA, respectively, for 72 h. The cell viability (%) was normalized with DMSO vehicle treated cells, and the result indicated that shh increases the chemo-resistant prostate cancer cell viability which is suppressed by PDE4D knockdown (*n* = 3, * *p* < 0.05, ** *p* < 0.01).

We and colleagues previously demonstrated that PDE4D inhibitor, Eggmanone, blocks the sonic hedgehog signaling and leads to cAMP elevation which in turn activates Protein kinase A, a negative regulator of the sonic hedgehog signaling [20]. To test if Eggmanone overcomes prostate cancer cell chemo-resistance via sonic Hedgehog signaling, we treated the chemo-resistant prostate cancer DU-145TxR and PC3-TxR cell with shh (200 ng/mL), shh (200 ng/mL) combined with 3 µM Eggmanone, shh (200 ng/mL) with siRNA control and shh (200 ng/mL) with siRNA PDE4D. The MTS assay showed that shh increases the proliferation of the chemo-resistant prostate cancer cells by approximately 22% for DU145-TxR and 37% for PC3-TxR (Figure 4A,B). This is consistent with previous reports that sonic hedgehog signaling promotes chemoresistance of cancer cells [27,28,29,30]. While Eggmanone treatment or PDE4D knockdown with siRNA dramatically attenuated the proliferation of the chemo-resistant prostate cancer cells in the presence of sonic Hedgehog ligand, suggesting that Eggmanone overcomes prostate cancer cell chemo-resistance via sonic Hedgehog signaling.

### 3.5. Treatment with the Combination of Eggmanone and Docetaxel Dramatically Overcomes Prostate Cancer Cell Chemo-Resistance

**Figure 5:**
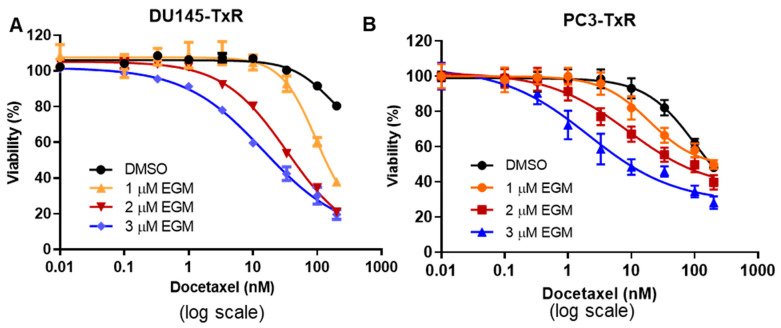
Treatment with the combination of Eggmanone and docetaxel dramatically overcomes prostate cancer cell chemo-resistance. (**A**) the chemo-resistant prostate cancer DU145-TxR cells were treated with both Eggmanone (1 µM, 2 µM and 3 µM) and docetaxel (0, 0.1, 0.33,1, 3.3, 10, 33.3, 100 and 200 nM) for 72 h. The control cell viability (no DTX) was designated as 100%. Cell viabilities were normalized to the control (no DTX) and cell viability at different DTX concentrations with 1 µM, 2 µM or 3 µM Eggmanone was plotted. The result showed that Eggmanone dose-dependently increased cytotoxicity of docetaxel to chemo-resistant DU145-TxR. (**B**) The chemo-resistant prostate cancer PC3-TxR cells were treated with both Eggmanone (1 µM, 2 µM and 3 µM) and docetaxel (0, 0.1, 0.33,1, 3.3, 10, 33.3, 100 and 200 nM for 72 h. The control cell viability (no DTX) was designated as 100%. Cell viabilities were normalized to the control (no DTX) and cell viability at different DTX concentrations with 1 µM, 2 µM or 3 µM Eggmanone was plotted. The result showed that Eggmanone dose-dependently increased cytotoxicity of docetaxel to chemo-resistant PC3-TxR.

DU145-TxR and PC3-TxR cells are very resistant to docetaxel treatment whereas Eggmanone can dramatically reduce the proliferation of these chemo-resistant prostate cancer cells. Next, we examined whether the combination of Eggmanone and docetaxel can further decrease cell proliferation in DU145-TxR and PC3-TxR cells. Eggmanone at concentrations of 1 µM, 2 µM and 3 µM in combination with docetaxel at various concentrations (0, 0.1, 0.33,1, 3.3, 10, 33.3, 100 and 200 nM) was administered to DU145-TxR and PC3-TxR cells. After 72 h incubation, the cells are subjected to MTS assay. The result indicated that Eggmanone increased the cytotoxicity of docetaxel to both DU145-TxR and PC3-TxR cells dose-dependently (Figure 5A,B). In comparison to docetaxel treatment alone (IC50 =161.5 nM), Eggmanone significantly decreased docetaxel’s IC50 s to DU145-TxR cells (94.84 nM for 1 µM Eggmanone, 34.16 nM for 2 µM Eggmanone and 14.58 nM for 3 µM Eggmanone) (Table 1). The similar result was also observed in PC3-TxR cells (Table 1). This data suggests that the combination of Eggmanone and docetaxel dramatically overcomes the prostate cancer cell chemo-resistance.

### 3.6. Eggmanone Attenuates Stem-Like Properties of Chemo-Resistant Prostate Cancer Cells

**Figure 6:**
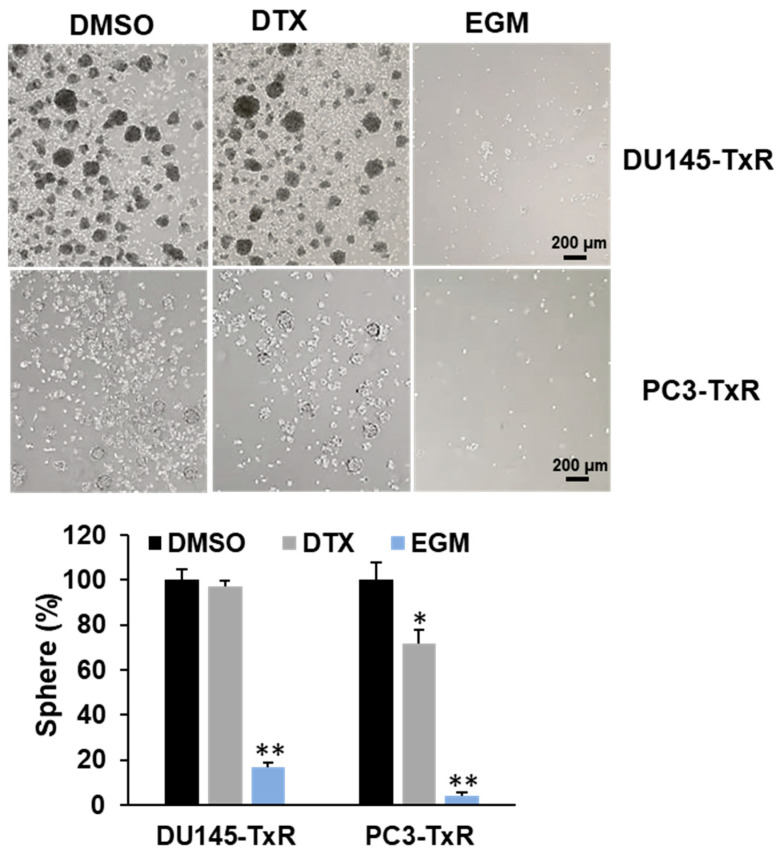
Eggmanone inhibits sphere formation in chemo-resistant prostate cancer cells.Sphere formation assay in chemo-resistant DU145-TxR and PC3-TxR cells treated with vehicle DMSO, 1 nM Docetaxel (DTX) and 3 µM Eggmanone (EGM). (* *p* < 0.05, ** *p* < 0.01).

**Figure 7:**
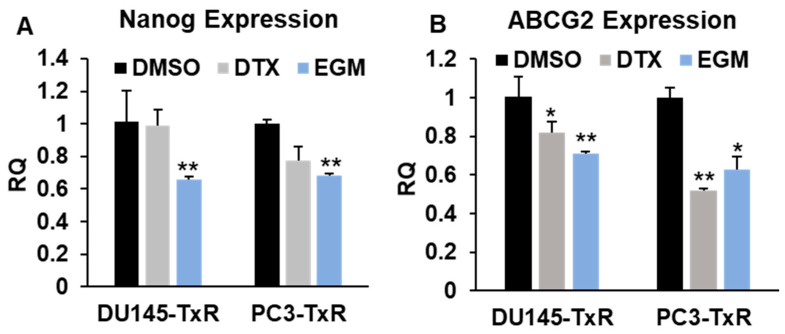
Eggmanone attenuates the expression of CSCs’ markers in chemo-resistant prostate cancer cells. (**A**) RT-PCR shows that mRNA of Nanog is statistically significantly down-regulated in chemo-resistant DU145-TxR and PC3-TxR cells treated with 3 µM Eggmanone (EGM). (**B**) RT-PCR shows that mRNA of ABCG2 is statistically significantly decreased in chemo-resistant DU145-TxR and PC3-TxR cells treated with 3 µM Eggmanone (EGM). RT-PC result was represented as mean relative quantity (RQ) ± SEM (*n* = 3, * *p* < 0.05, ** *p* < 0.01).

Cancer stem cells (CSCs) are believed to play an important role in cancer initiation, metastasis and chemo-resistance [31,32]. We, therefore, examined whether Eggmanone exerts effects on CSCs by the sphere-formation assay, a commonly used in vitro method for studying CSCs’ properties [33]. In brief, the chemo-resistant prostate cancer cells were plated on low-attached 6-well-plate for suspension culture of 14 days, and the sphere-forming capacity was assessed by the number of colonies in size larger than 50 µm diameter. As shown in Figure 6, docetaxel treatment did not alter the sphere number in DU145-TxR but modestly decreased the sphere number in PC3-TxR by approximately 28%. In contrast, Eggmanone treatment led to 83% and 96% of decrease in the sphere numbers of DU145-TxR and of PC3-TxR cells, respectively. Furthermore, we examined whether Eggmanone treatment actually alters the expression of CSCs markers in the chemo-resistant prostate cancer cells. Nanog and ABCG2 are two marker genes known for prostate CSC [34,35,36,37], and our RT-PCR result demonstrated that Eggmanone can dramatically down-regulated the expression of both Nanog and ABCG2 in DU-145-TXR and PC3-TxR cells (Figure 7). In summary, Eggmanone treatment attenuates stem-like properties of the chemo-resistant prostate cancer cells.

## 4. Discussion

PDE4 is an important intracellular enzyme in the cAMP/CREB signaling pathway which is known to be implicated in many biological functions such as cell cycle, cell survival and cell differentiation [38,39]. In recent years, PDE4 subtype, PDE4D, has been identified as a cancer-promoting molecular that represents a novel target in many types of cancer including as lung cancer, melanoma, colorectal cancer, leukemia, colon cancer and glioma [10,11,12,13,14,40,41]. In human CRPC prostate cancer, PDE4D is highly overexpressed [11] and inhibition of PDE4D reduces the growth of both prostate cancer cells in vitro and in vivo [11,19]. Nevertheless, all the current studies of PDE4D in prostate cancer have focused on the non-chemo-resistant prostate cancer, and no PDE4D studies in the more-advanced chemo-resistant prostate cancer have been reported to date. In this study, we found that PDE4D is the only PDE4 subtype with highly upregulated expression in the chemo-resistant prostate cancer cells in comparison to the chemo-sensitive prostate cancer cells. PDE4D inhibitor Eggmanone was found to significantly decrease the invasion and proliferation of the chemo-resistant prostate cancer cells, and treatment with the combination of Eggmanone and docetaxel dramatically overcomes prostate cancer cell chemo-resistance. The hedgehog signaling has been reported to promote cancer cell chemoresistance [27,28,29,30], and we and colleagues have previously demonstrated that PDE4D inhibitor Eggmanone elevates cAMP which in turn activates Protein kinase A, leading to the sonic hedgehog signaling inhibition [20]. In consistence, the knockdown of PDE4D with siRNA induced similar effects on proliferation of chemo-resistant prostate cancer cells. In addition, Hedgehog pathway has been reported to be implicated in regulation of cell invasion in pancreatic cancer and gastric cancer [42,43], and the invasion reduction by Eggmanone in prostate cancer cells may be attributed to inhibition of Hedgehog signaling.

CSCs are associated with chemo-resistance in many types of cancer [44], including breast cancer [45], glioblastoma multiforme [46] and prostate cancer [47]. Eggmanone treatment dramatically down-regulated CSC markers Nanog and ABCG2 as well as inhibited the sphere formation in the chemo-resistant prostate cancer DU-145-TXR and PC3-TxR cells. These results suggest that Eggmanone may attenuate the proliferation and induce the cell death of the chemo-resistant prostate cancer cells by targeting prostate cancer CSCs. This result is consistent with a recent report that a small molecule CG500354 induced growth arrest and attenuated stemness in glioblastoma multiforme CSCs by inhibiting PDE4D [48].

Eggmanone is a potent and selective allosteric PDE4D inhibitor found an in vivo zebrafish phenotype-based screening [20], and our recent study indicated that Eggmanone displays favorable drug pharmacokinetics in rats [24]. Hence, Eggmanone could be a novel therapeutic agent for overcoming the chemo-resistance of prostate cancer.

## Figures and Tables

**Table 1:** Docetaxel IC50 to chemo-resistant prostate cancer cells is decreased along with elevation of Eggmanone in a dose dependent way.

	DTX IC50 (nM)			
Cell Line	DMSO	1 µM EGM	2 µM EGM	3 µM EGM
DU145-TxR	161.5	94.84	34.16	14.58
PC3-TxR	103	19.41	8.85	2.11

## Data Availability

Not applicable.

## Supplementary Materials

The following are available online at https://www.mdpi.com/article/10.3390/biomedicines9050538/s1, Figure S1: Effects of PDE4D knockdown on invasion of the chemo-resistant prostate cancer DU145-TxR and PC3-TxR cells, Figure S2: Cell invasion and migration assay in the chemo-sensitive DU145 and PC3 cells treated with DMSO or 3 µM Eggmanone, Figure S3: PDE4D knockdown with siRNA induces death of chemo-resistant prostate cancer cells, Figure S4: Cell apoptosis assay in the chemo-resistant DU145-TxR and PC3-TxR cells treated with DMSO, or 3 µM Eggmanone for 24 h, Table S1: The primer sets used in the study.
