# Supplementary material for: Eggmanone Effectively Overcomes Prostate Cancer Cell Chemoresistance
Source: Biomedicines. 2021 May 12;9(5):538. doi: 10.3390/biomedicines9050538 (PMC8151738; doi:10.3390/biomedicines9050538)
Supplement: Supplementary file 1 [file biomedicines-09-00538-s001.zip › biomedicines-1142886-supplementary.pdf]

biomedicines

MDPI

Article

Eggmanone Effectively Overcomes Prostate Cancer Cell Chemoresistance

Chen Xie [1], Pen-Jen Lin [2] and Jijun Hao [1,2,*]

Supplementary materials

Table S1. The primer sets used in the study.

Genes	Oligonucleotides
Human GAPDH	5'-GGTGTGAACCATGAGAAGTATGA-3' (forward)<br>5'-GTCCTTCCACGATACCAAAG-3' (reverse)
Human PDE4A	5'-TACAGCCCATGTCCCAAATC-3' (forward)<br>5'-GAGCTCTTCTTGATCGGTCTTC-3' (reverse)
Human PDE4B	5'-CAGACCTGAAGACAATGGTAGAA-3' (forward)<br>5'-GACCTGAATGCGATCGGTATAG-3' (reverse)
Human PDE4C	5'-GGGCAACGACAACAACAATAA-3' (forward)<br>5'-CCTACATCCAGAGACACCAAAG-3' (reverse)
Human PDE4D	5'-TTACCAGTAGGAGGAGGATGAG-3' (forward)<br>5'-AGCTCACTGAACCACACTATTC-3' (reverse)
Human ABCG2	5'- GTGTGTCTGGAGGAGAAAGAAA-3' (forward)<br>5'- GCTTGAGTCTAAGCCAGTTGTA-3'(reverse)
Human Nanog	5'-TTTGGAAGCTGCTGGGGAAG-3' (forward)<br>5'-GATGGGAGGAGGGGAGAGGA-3' (reverse)

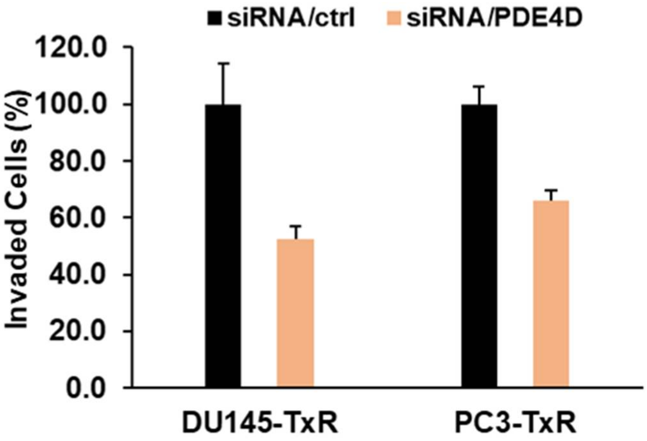

Figure S1. Effects of PDE4D knockdown on invasion of the chemo-resistant prostate cancer DU145-TxR and PC3-TxR cells. Results were determined by the modified Boyden chamber assay in a 24-Multiwell Insert System (8 μM membrane, BD Biosciences) coated with Matrigel. The cells were treated for 72 hours, and the invading cell percentages were normalized to the DMSO vehicle treated controls.

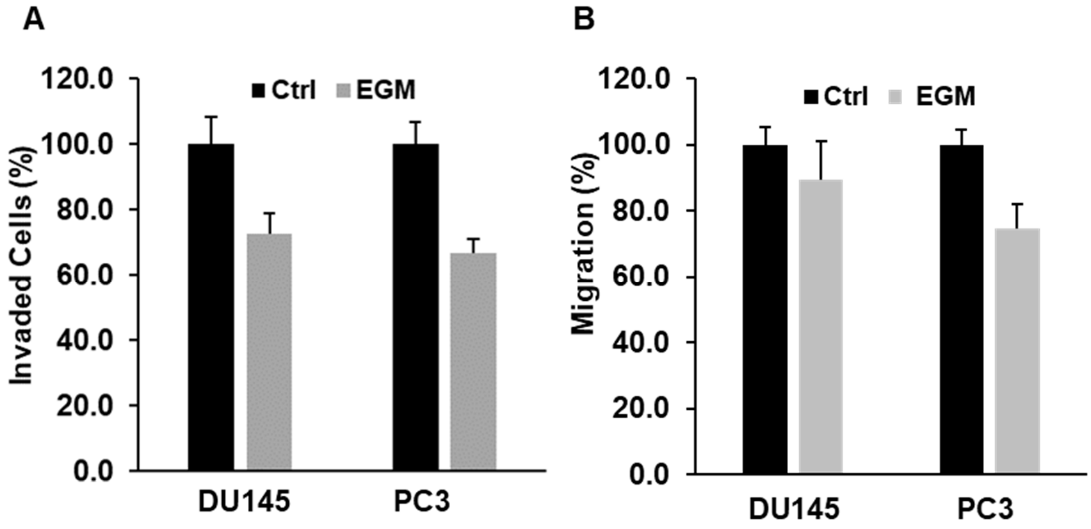

Figure S2. Cell invasion (A) and migration (B) assay in the chemo-sensitive DU145 and PC3 cells treated with DMSO or 3 μM Eggmanone.

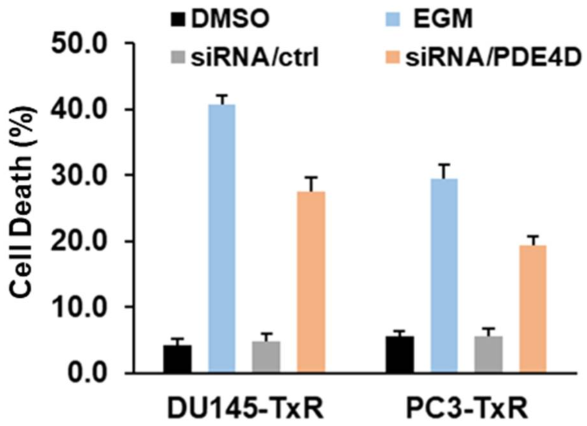

Figure S3. PDE4D knockdown with siRNA induces death of chemo-resistant prostate cancer cells. The chemo-resistant prostate cancer DU145-TxR and PC3-TxR cells were treated with DMSO vehicle, 3μM Eggmanone, siRNA control and siRNA PDE4D for 72 hours, the cells were harvested for cell death assay using Trypan Blue staining. The result showed that 3μM Eggmanone and siRNA knockdown of PDE4D significantly reduced cell deaths in chemo-resistant prostate cancer cells.

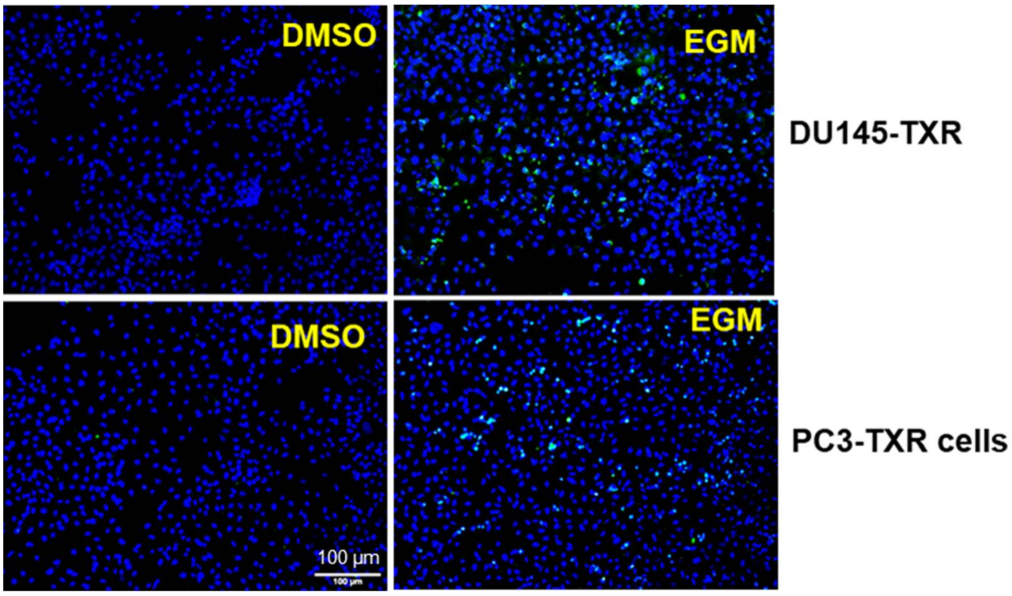

Figure S4. Cell apoptosis assay in the chemo-resistant DU145-TxR and PC3-TxR cells treated with DMSO, or 3 μM Eggmanone for 24 hours. The apoptotic cells were detected with the CellEvent Caspase-3/7 Green Detection Reagent (ThermoFisher) and DAPI was used for counterstaining.
